# Evaluation of Clustering Techniques to Predict Surface Roughness during Turning of Stainless-Steel Using Vibration Signals

**DOI:** 10.3390/ma14175050

**Published:** 2021-09-03

**Authors:** Issam Abu-Mahfouz, Amit Banerjee, Esfakur Rahman

**Affiliations:** School of Science, Engineering, and Technology, Penn State Harrisburg, Middletown, PA 17057, USA; aub25@psu.edu (A.B.); aer15@psu.edu (E.R.)

**Keywords:** clustering, prediction, surface roughness, turning, vibration

## Abstract

In metal-cutting processes, the interaction between the tool and workpiece is highly nonlinear and is very sensitive to small variations in the process parameters. This causes difficulties in controlling and predicting the resulting surface finish quality of the machined surface. In this work, vibration signals along the major cutting force direction in the turning process are measured at different combinations of cutting speeds, feeds, and depths of cut using a piezoelectric accelerometer. The signals are processed to extract features in the time and frequency domains. These include statistical quantities, Fast Fourier spectral signatures, and various wavelet analysis extracts. Various feature selection methods are applied to the extracted features for dimensionality reduction, followed by applying several outlier-resistant unsupervised clustering algorithms on the reduced feature set. The objective is to ascertain if partitions created by the clustering algorithms correspond to experimentally obtained surface roughness data for specific combinations of cutting conditions. We find 75% accuracy in predicting surface finish from the Noise Clustering Fuzzy C-Means (NC-FCM) and the Density-Based Spatial Clustering Applications with Noise (DBSCAN) algorithms, and upwards of 80% accuracy in identifying outliers. In general, wrapper methods used for feature selection had better partitioning efficacy than filter methods for feature selection. These results are useful when considering real-time steel turning process monitoring systems.

## 1. Introduction

Surface finish is one of the most important quality measures that affect the product cost and its functionality. Examples of functionality characteristics include tribological properties, corrosion resistance, sliding surface friction, light reflection fatigue life, and fit of critical mating surfaces for assembly. It is normally specified for a certain application in order to achieve the desired level during machining. Factors that may affect the surface finish in machining such as the machining parameters, hardness of workpiece material, selection of cutting tool and tool geometry, must be carefully selected to obtain desired product quality. A review on the effective and accurate prediction of surface roughness in machining is presented in [1].

Several attempts have been made for modeling and predicting surface roughness in the turning of steel machine components. The design of experiment approaches, such as the Taguchi method, involves the conduction of systemic experiments and collection and performing comparative analysis of the data [2]. In [3], the Taguchi method was applied for turning process parameter optimization to obtain the least vibration and surface roughness in dry machining of mild steel using a multilayer coated carbide insert (TiN-TiCN-Al_2_O_3_-ZrCN). Experimental investigation approaches used regression analysis models that relate machining variables with surface roughness [4]. A force prediction regression model was developed [5] for finish turning of hardened EN31 steel (equivalent to AISI 52100 steel) using hone edge uncoated cubic boron nitride (CBN) insert for better performance within a selected range of machining parameters. The developed regression models could be used for making predictions for the forces and surface roughness for energy-efficient machining. Fitness quality of the data was analyzed using the ANOVA method. The effect of the turning process parameters in addition to the tool nose radius on the surface roughness of AISI 10 steel was investigated in [6] by using Design of Experiment (DOE) and the Response Surface Methodology (RSM). The constructed surface contours were used to develop a mathematical prediction model for determining the optimum conditions for a required surface roughness. In [7], the nature of vibrations arising in the cutting tool at different cutting conditions has been investigated. It has been observed that the root mean square (RMS) amplitude of the vibration response along the main cutting direction was mixed. The feed direction vibration component has a similar response to the change in the workpiece surface roughness, while the radial and cutting vibration components have a more coherent response to the rate of flank wear progression throughout the tool life. A surface finish quality study [8] compared the effects of tool geometries and tool materials in the turning of three engineering steels, namely, hardened 410, PH13-8Mo, and 300M, two stainless steels and one high strength steel. The investigation aimed at identifying the optimum feed rate and cutting speed for optimum cutting quality. An expert system is developed in [9], based on the fuzzy basis function network (FBFN) to predict surface finish in ultra-precision turning. An approach for automatic design of rule base (RB) and the weight factors (WFs) for different rules is developed using a genetic algorithm based on error reduction measures. In [10], the Artificial Neural Network (ANN), response surface method (RSM), Desirability function approach (DF), and the Non-dominated Sorting Genetic Algorithm (NSGA-II) were used to model the surface roughness and cutting force in finish turning of AISI 4140 hardened steel with mixed ceramic tools. It was found that the NSGA-II coupled with ANN to be more efficient than the DF method and allowed for better prediction of surface roughness and cutting forces than the other methods. A digital twin model for surface roughness prediction that implements sensor fusion in the turning process was presented in [11]. This system combined preprocessed vibration and power consumption signals with cutting parameters for feature vector construction. The principal component analysis and support vector machine were used for feature fusion and surface roughness prediction, respectively. The influence of machining parameters on the surface finish of medical steel in the turning process using an adaptive-neuro-fuzzy system (ANFIS) was investigated in [12]. Surface roughness parameters were optimized by the use of the ant colony method.

The objective of this work is to determine whether it is possible to treat the prediction of surface finish in turning of steel samples as an unsupervised clustering problem based on features extracted from vibration data. The specific objectives are:

1. Identification of a smaller subset of features from the feature-rich vibration data that can be used as a predictor of surface roughness. This is achieved by employing and comparing various feature selection methods.
2. Unsupervised clustering of experimentally obtained data with features identified using feature selection techniques. The clustering results are then compared to measured values of surface roughness (R_a_). This will then be used a basis to identify optimal cutting conditions (feed, speed and depth of cut) to produce the best surface finish.
3. Identification of noisy data based on extracted features using various noise-resistant unsupervised clustering methods. In practice, datasets may contain outliers and it is important to use clustering techniques that identify such outliers and cluster the rest of the dataset meaningfully.
4. Comparison of different methods for feature selection and unsupervised clustering.

## 2. Experiment

**Figure 1:**
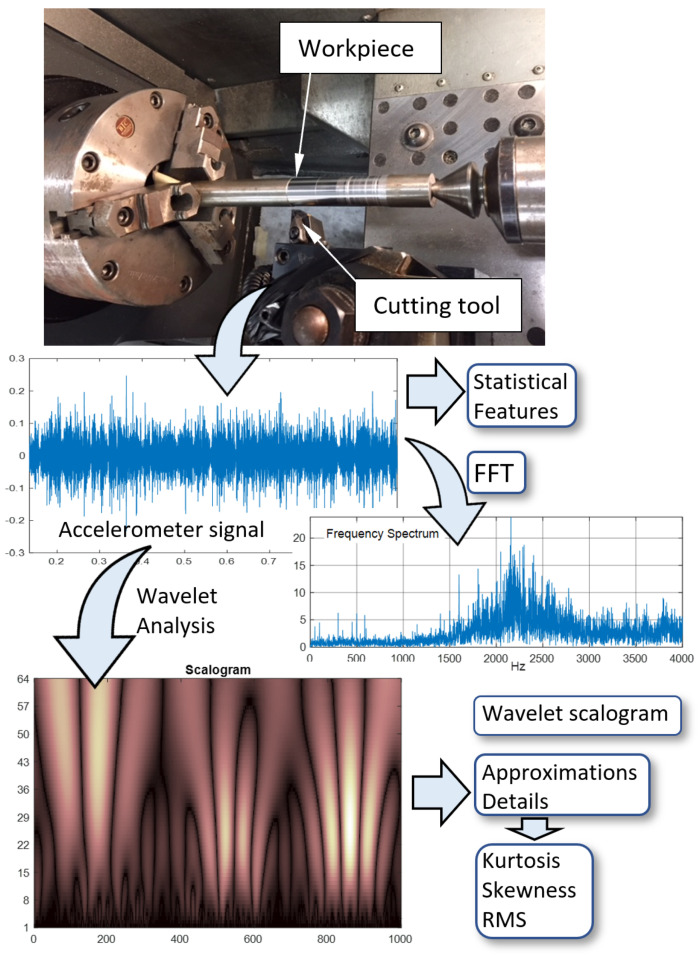
Turning test and vibration signal processing scheme.

Figure 1 shows the experimental setup for the turning process. All machining cuts were performed on austenitic stainless steel (304) bar stocks with 23.79 mm diameter. Properties of the stainless steel bar stock used in this research are included in Appendix A (Table A1, Table A2 and Table A3). A model WNMG 432-PM 4325 Sandvik Coromant turning inserts were used for all turning passes. A fresh cutting edge free of any signs of wear or fracture is ensured for each turning run. As shown in Figure 1, the work piece is supported at its free end by using a life turning center on the tailstock. This will give more stability and reduce oscillations during machining.

**Figure 2:**
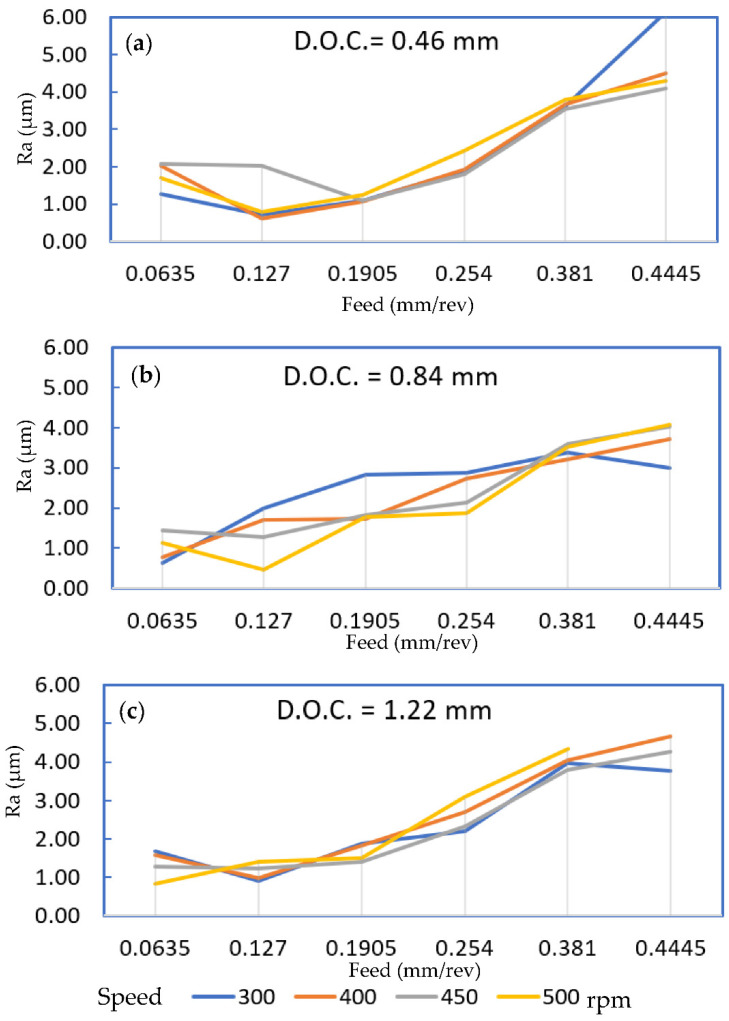
Averaged surface roughness (Ra) in µm at four speeds for (**a**) D.O.C. = 0.46 mm, (**b**) D.O.C. = 0.84 mm, and (**c**) D.O.C. = 1.22 mm.

A model 607A61 ICP accelerometer (Integrated Circuit Piezoelectric (ICP) is a registered trademarks of PCB Piezotronics, Inc., Depew, NY, USA) with a sensitivity of 100 mV/g was mounted on the tool shank with orientation to measure vibration signals along the cutting (tangential) direction of the bar stock. Ninety (90) combinations of turning process parameters were based on three depths of cut (D.O.C.; 0.46mm, 0.84 mm, and 1.22 mm), five speeds (300, 350, 400, 450, and 500 rpm), and six feed rates (0.064, 0.127, 0.19, 0.254, 0.381, and 0.445 mm/rev). These cutting conditions were selected for fine machining, and for each combination of cutting conditions, the work piece was machined for a 25 mm long turning pass. Additionally, for each set of turning process parameter combinations, accelerometer signals were recorded using an NI-9230 C Series Sound and Vibration Input Module via a National Instruments CompactDAQ data acquisition system (ni, Austin, TX, USA). The surface roughness parameter (Ra), in µm, was measured using the Handysurf E-35A for each run along the feed direction and averaged for each cutting parameter combination. A summary of the averaged surface roughness measurements is shown in Figure 2. The missing data point in Figure 2c, for D.O.C = 1.22 mm, feed rate = 0.4445 mm/rev, and speed of 500 rpm, was omitted since these conditions resulted in a very rough surface due to unstable chatter during the turning process.

## 3. Signal Processing

**Figure 3:**
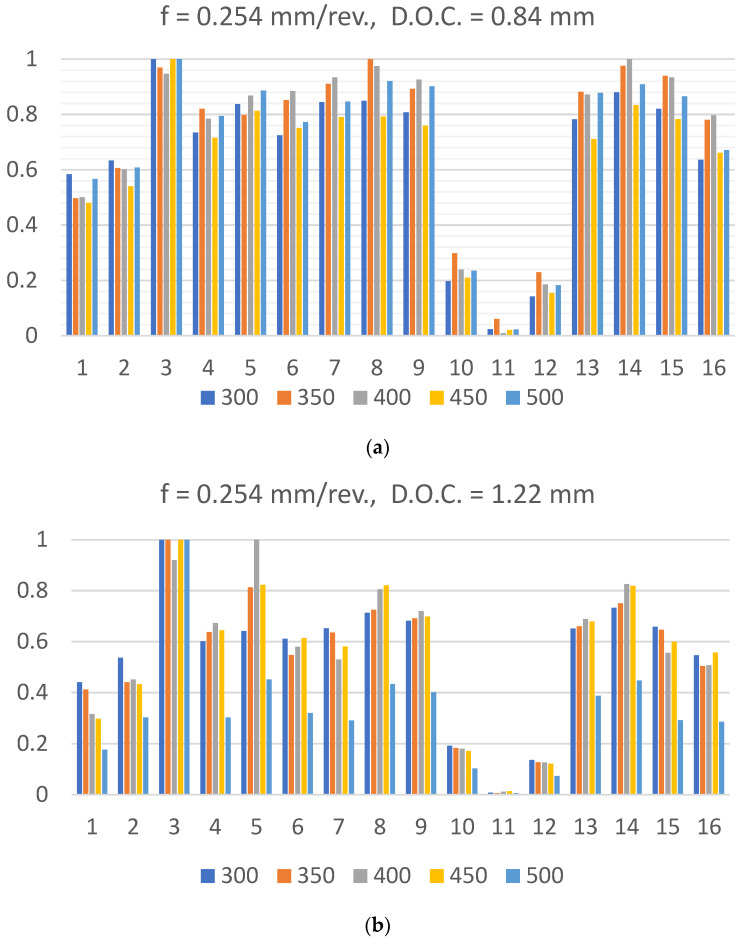
Sample FFT averaged 16 bands for a feed rate = 0.254 mm/rev, and for different speeds at: (**a**) D.O.C. = 0.84 mm, and (**b**) D.O.C. = 1.22 mm.

Time series signatures of the vibration signals were processed for dimensionality reduction and feature extraction using statistical, frequency, and time-frequency analysis techniques. Figure 3 shows two samples of 16 averaged and normalized Fast Fourier Transform (FFT) frequency bands. For the time-frequency analysis, two continuous wavelet transform (cwt) functions, the Coiflet4 and the Mexican Hat wavelets, were applied to vibration time signals. Sixty four (64) averaged scales of the scalogram were calculated as features of interest.

**Figure 4:**
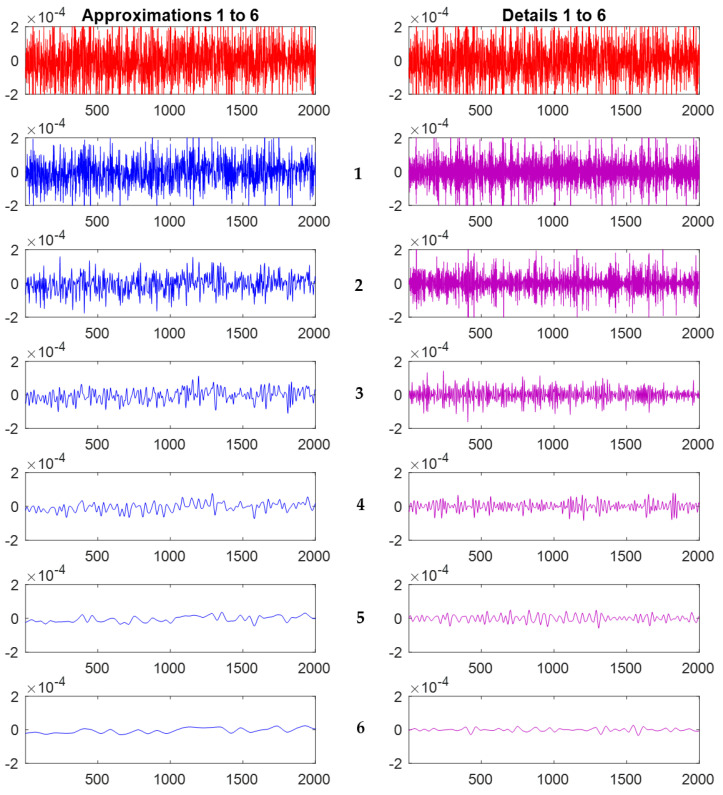
Sample wavelet decomposition for the first 6 out of 10 approximations (**left**) and details of the vibration time signal (**right**) at 300 rpm, feed = 0.0635 mm/rev, and D.O.C. = 0.46 mm.

**Figure 5:**
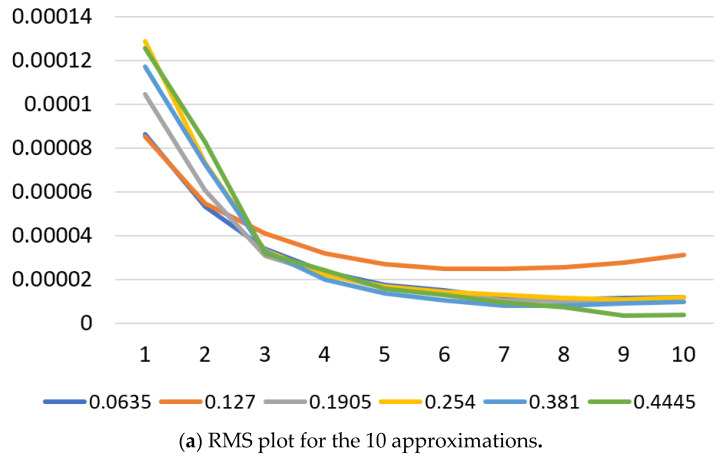

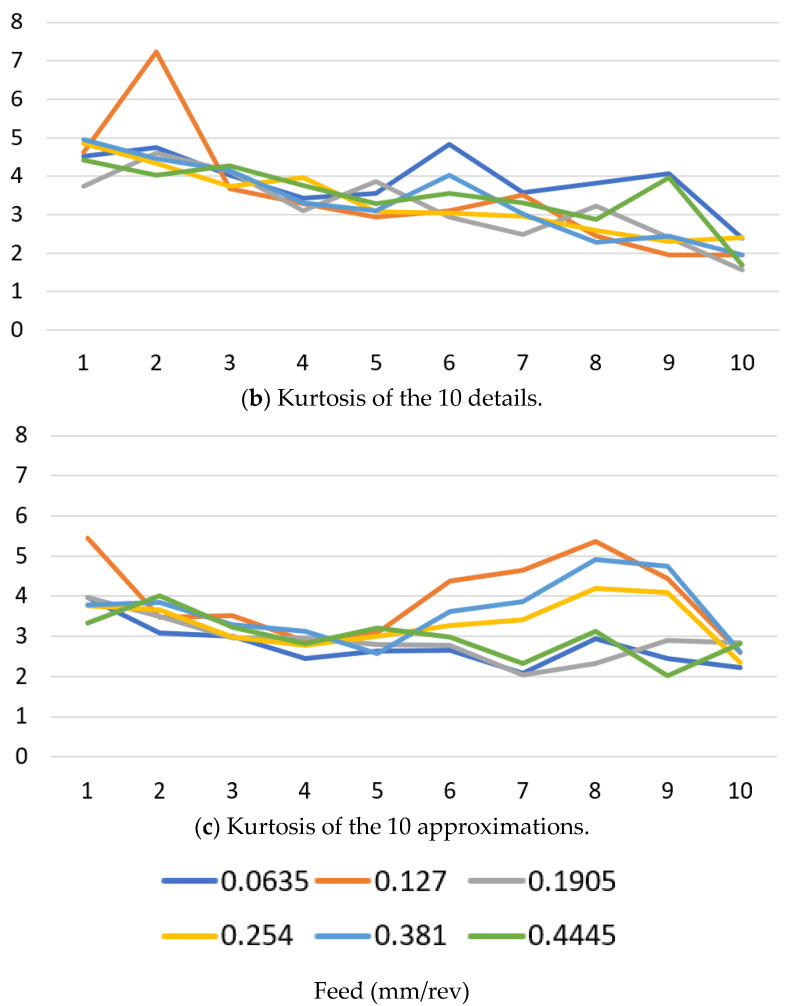
Sample results for 10 wavelet approximation and details at 400 rpm and 1.22 mm D.O.C.

The wavelet transform decomposes the original signal successively into lower resolutions. Sample approximations and details for the first six decomposition levels, out of the 10 levels calculated for this study, are shown in Figure 4. These signals were calculated using the (cwt) MATLAB (The MathWorks, Inc., Natick, MA, USA) function and the Coiflet4 wavelet. The top signal in red is the original vibration signal. Statistical parameters are calculated for the raw vibration signals and for each one of the 10 decomposed signals of the approximations and details. These parameters include the mean, RMS, standard deviation, kurtosis, and skewness. These are used as features in this study following successful implementation in previous work by the authors [13,14]. Sample results of the RMS and kurtosis calculations for the approximations of the wavelet decomposition are shown in Figure 5.

As can be seen from these sample results, patterns of a separable nature can be observed by some features in some regions of the turning process parameters but are not as clear in other regions. Therefore, using more advanced clustering techniques for feature grouping and selection is inevitable in this case of highly complex and nonlinear steel turning process. The following sections aim at detailing the unsupervised clustering techniques and evaluating their ability to predict the surface finish of the turned stainless steel parts as implemented in this research.

## 4. Methods

Machine learning methods have been used in the identification of optimal machining parameters. These include classification algorithms, both supervised or unsupervised, regression models and deep learning models. Classification techniques are used to categorize data defined in feature space into known discrete classes. There are two general approaches for classification—supervised clustering or supervised learning trains a classifier and therefore needs training data. The classifier in the training step is set up by examining surface roughness data that are already classified with the correct roughness class label (Table 1). This trained classifier can then be used to predict the class of unlabeled data (data for which surface finish measurements are not available). The other approach is unsupervised clustering, which does not require training a classifier in the sense that it directly predicts the class of unlabeled data by grouping together self-similar datapoints based on a similarity or dissimilarity measure. Regression models are used for prediction, usually a continuous output variable. In this case, given the features that represent the accelerometer signals during turning, a regression model can be used to predict the value of the average surface roughness (Ra). Deep learning methods use artificial neural networks that are trained to identify patterns in input–output data. Like supervised learning, deep learning also needs training data to tune the model and test data to identify patterns in unlabeled data. One of the major drawbacks in using supervised and/or deep learning models and regression models is that one requires a large dataset to ensure the training phases produce a meaningful classifier. In this study, the size of the dataset is not large; however, the feature set is large, and therefore the analysis lends itself well to unsupervised classification or clustering.

### 4.1. Feature Selection

Feature selection can be understood as finding the “best subset of features or a combination of features” that leads to the most optimum classification of the dataset. In the absence of training data, the most optimum classification can be estimated by comparing using the ground truth (preassigned three-cluster labels from surface roughness data in this case). Feature selection techniques can be partitioned into three basic methods [15]: (1) wrapper-type methods which use classifiers to score a given subset of features; (2) embedded methods, which inject the selection process into the learning of the classifier; and (3) filter methods, which analyze intrinsic properties of data, ignoring the classifier. Most of these methods can perform subset selection and ranking. Generally, the subset selection is always supervised, while in the ranking case, methods can be supervised or not. In this paper, we use six feature selection methods from the Feature Selection Library (FSLib 2018), a publicly available MATLAB library for feature selection [16]. These feature selection methods are listed in Table 2 below.

The performance of MCFS can be compared to LS since they are both unsupervised filter methods, while the performance of UFSOL and DGUFS can be compared since they are both unsupervised wrapper methods for feature selection. For more details, the reader is referred to [16].

### 4.2. Data Analysis

Clustering or classification based on raw data implies working in a high dimensional space, especially for time series data collected in our study at fast sampling rates. Due to possible outliers in the data, we use a robust version of the fuzzy c-means clustering algorithm as the data clustering technique. This is then compared to three other unsupervised techniques: (1) kernel clustering using radial basis function kernels and kernel k-means, (2) spectral clustering, and (3) spatial density-based noise-resistant clustering. Clustering has been used in the literature to cluster data from manufacturing processes for tool condition monitoring and to identify specific patterns for parameter optimization. Clustering techniques are applied to wavelet features of force and vibration signals in a high-speed milling process [17]. It was shown clustering can be applied to fault diagnosis and tool condition monitoring. Process modeling of an abrasive water-jet machining process for the machining of composites was performed using a fuzzy logic and expert system with subtractive clustering for the prediction of surface roughness [18]. Unsupervised clustering and supervised classification have been successfully used to predict surface finish in turning [13]. To the best of our knowledge, there has not been any work in using unsupervised classification to identify optimal parameters for the turning of steel samples.

#### 4.2.1. Fuzzy Clustering

In clustering, each datapoint belongs to a specific cluster; however, in fuzzy clustering, the notion of partial-belongingness of datapoints to clusters is introduced. A data object *x_j_* has a membership of *u_ij_* in the interval [0,1] in a cluster *i*, which can be defined as the partial belongingness of the datapoint to that cluster, subject to the constraint that the sum of memberships across all clusters is unity and the contribution of memberships of all data points to any particular cluster is always less than the size of the dataset *n*.

(1) $$\sum_{i\;=\;1}^{k}u_{ij}=1;\;\;\;0\;<\;\sum_{j\;=\;1}^{n}u_{ij}\;<\;n$$

The fuzzy squared-error-based objective function is the modified fuzzy least-squares estimator function given by

(2) $$J=\sum_{i\;=\;1}^{c}\sum_{j\;=\;1}^{n}u_{ij}^{m}\left\|x_{j}-v_{i}\right\|$$

The exponent *m,* called the fuzzifier, determines the *fuzziness* of the partition and ‖ ‖ is the distance measure between datapoint *x_j_* and cluster prototype *v_i_* of cluster *i*. The prototypes *v_i_* are initialized, either randomly or procedurally. The prototypes are then refined using an alternation optimization procedure. At each optimization step, the partition memberships and the prototypes are updated, until a pre-defined stopping criterion is met, such as when prototypes have stabilized. While the requirement that the sum of memberships of a datapoint across all clusters be unity is an attractive property when the data have naturally overlapping clusters, it is detrimental when the data have outliers. In the latter case, the outliers (like good datapoints) will have significantly high membership values in some clusters, therefore contributing to incorrect parameter estimates of the cluster prototype.

Noise-resistant versions of fuzzy clustering define a separate cluster called the *noise* cluster using a prototype which is equidistant from all datapoints [19,20,21]. This noise cluster allows the total membership of a datapoint in all the “good” clusters to be less than unity; the difference is made up by its membership value in the noise cluster. This also allows outliers to have small membership values in good clusters. The objective function to be minimized is,

(3) $$J=\sum_{i\;=\;1}^{c}\sum_{j\;=\;1}^{n}u_{ij}^{m}\left\|x_{j}-v_{i}\right\|+\sum_{j\;=\;1}^{n}\delta^{2}(1-\sum_{i\;=\;1}^{c}u_{ij}{)}^{m}$$

Noise distance is defined as a large threshold distance which can either be assigned arbitrarily based on data scales or can be tuned iteratively during clustering. Assuming that a fraction *λ* of data points might be outliers, a way to set noise distance is to tune the value of *λ* by using a parallel alternating optimization procedure to minimize intra-cluster distance and maximize inter-cluster distances with different values of *λ*. The noise distance was initially defined as a function of the mean squared point-prototype distances as

(4) $$\delta^{2}=\frac{\lambda }{cn}\sum_{i\;=\;1}^{c}\sum_{j\;=\;1}^{n}\left\|x_{j}-v_{i}\right\|$$

In this paper, the noise clustering algorithm is implemented with *λ* = 0.05 which translates to 5% of data points can be potential outliers. The fuzzifier *m* is chosen to be 2.0. There is a theoretical foundation for such a generalization [22]. However, in practice *m* = 2 has seem to work better than other choices. In [23], rail cracks were identified from acoustic emission signals and noise clustering. In a related work, structural damage in truss structures was detected from finite element modeling data and the noise clustering-based swarm optimization technique [24]. Both studies use a threshold-based noise distance and a robust *k*-means clustering algorithm for detection. The noise-resistant fuzzy clustering algorithm here will be referred to as NC (Noise Clustering) for the reminder of this paper.

#### 4.2.2. Spectral and Kernel Clustering

These algorithms are a class of graph-based kernel methods that use the top eigenvectors and eigenvalues of either the proximity matrix or some variant of the distance matrix. These algorithms project data into a lower dimensional eigenvector subspace, which generally amplifies the block structure of the data. Multiway spectral algorithms use partitional algorithms to cluster the data in the lower *k*-dimensional eigenvector space, while recursive spectral clustering methods produce a two-cluster partition of the data followed by a recursive split of the two clusters, based on a single eigenvector each time. The bipartition is recursively partitioned until all *k*-clusters are discovered [25]. In this paper, we used the standard spectralcluster function in MATLAB’s Statistical and Machine Learning Toolbox, and refer to the algorithm as SC (Spatial Clustering).

Other kernel-based clustering algorithms nonlinearly transform a set of complex and nonlinearly separable patterns into a higher dimensional feature space in which it might be possible to separate these patterns linearly [26]. Kernel-based approaches are known to be resistant to noise and outliers and include such methods as Support Vector Clustering (SVC) using radial basis functions [27] and fuzzy memberships [28]. These optimize the location of a set of contours as cluster boundaries in the original data space by mapping back the smallest enclosing sphere in the higher dimension feature space. The original data are mapped to a new *d*-dimensional space by implementing a transductive data wrapping using graph kernels, and the mapped data are used as the basis for a new affinity matrix [29]. The noise points are shown to map together as one compact cluster in the higher dimensional space and other clusters become well separated. In this paper, we use the Guassian (RBF) kernel and the kernel *k*-means as the two kernel-based clustering algorithms tested as presented in [30]. These will, respectively, be referred to as RBF-KC (Radial Basis Function-Kernel Clustering) and KKM-KC (Kernel k-Means-Kernel Clustering).

#### 4.2.3. Spatial Clustering

Spatial clustering methods such as the very popular Density-Based Spatial Clustering Applications with Noise (DBSCAN) use a density-based approach to find arbitrarily shaped clusters and outliers (noise) in data [31]. The algorithm is simple to use and assumes the data occupy regions of varying densities in the feature space. It uses two parameters that can be easily tuned. In this paper, we use the dbscan function from MATLAB’s Statistical and Machine Learning Toolbox. The algorithm clusters the datapoints based on a threshold for a neighborhood search radius epsilon and a minimum number of neighbor minpts required to identify a core point.

## 5. Results

The dataset is composed of 84 experiments and each experiment has 213 total features, as listed in Table 2 (not including the class labels). The attributes of the dataset are of different types. Distance measures ‖ ‖ used in unsupervised clustering are sensitive to certain types of data and require them to be formatted properly to give the best optimal solution. Therefore, there is a need for a preprocessing step where the data can be transformed from one type to another or can be scaled to a specific range. In this paper, data values are normalized to lie in the range of 0 to 1. In a related work [14], the effect of transformation (nominal feature values are converted to numeric values), feature scaling with mean normalization (all features have a range of values, from −1 to 1), and normalization (all numeric values are normalized to lie in the range of 0 to 1) were estimated. It was found that normalization of all values produces the greatest effect on accuracy of the classification process. However, unlike the previous work, nominal value features (depth of cut, speed, and feed rate) are not used, nor are class labels as features in the clustering process, and therefore transformation and feature scaling do not apply. After a simple trial with three distance measures (Euclidean, Mahalanobis, and Manhattan), it was found that the Euclidean norm provided the best results and is the only distance measure used in this study.

Dimensionality reduction to decrease computational load and to increase predictive accuracy is the primary reason for employing feature selection prior to clustering or any meaningful pattern recognition procedure. The full set of 213 features will not produce optimal clustering performance because some of the features might be highly correlated, redundant, or simply unrelated in determining the predictive variable, in this case the surface roughness label. In the first preprocessing step, six feature selection techniques included MATLAB’s FSLib2021 are used and the results of feature selection are shown in Table 3.

RFE produces the most drastic reduction in the feature set size compared to the baseline ReliefF. It will be shown later that this happens with very little decrease in performance of any of the clustering algorithms. The filter type methods (LS and MCFS) result in larger feature sets than the wrapper-type methods (DGUFS and UFSOL). The reader is reminded that since these are unsupervised, they tend to retain many of the features that are deemed redundant by the supervised methods.

Each of the five algorithms (NC, SC, RBF-KC, KKM-KC, and DBSCAN) are implemented with each of the feature selection methods. Two of the feature selection methods (RFE and Relief-F) are supervised methods and therefore use the class labels. It therefore makes sense to compare RFE feature-based clustering to Relief-F feature-based clustering. A comparison of the other feature selection methods which are unsupervised (LS, MCFS, DGUFS and UFSOL) will require using class labels post hoc. The 83 cases, i.e., the combination of cutting conditions (referred to as instances for the remainder of the paper) are assigned their class labels after partitions are obtained. Consider an illustrative case: one of the clusters in the three-cluster partition has a total of 26 instances—7 instances with cluster label 1, 14 instances with cluster label 2 and 5 instances with cluster label 3. Instances not included in the three-cluster partition will be considered outliers and will be assigned a label of 0. Assume this illustrative cluster has two outlier instances. Quantification of misclassification by defining the following post hoc measures can be discussed and explained using this illustrative case.

*Accuracy* is defined as the ratio of the total number of correctly assigned instances to the total number of instances. For this it is assumed that the majority class label is the class label of a particular cluster. Illustrative case: assume that the cluster representing class label 2 has 12 misclassified instances and 14 correctly classified instances. If there were to be 18 correctly identified instances in the second cluster and 22 correctly identified instances in the third cluster, the accuracy of the partition is (14 + 18 + 22)/83 = 0.65.

*Precision* is used to determine the correctness of the partitions. *Recall* is used to quantify the completeness of the partitions. The Precision and Recall measures are calculated for each partition, one partition at a time. Precision for a class is calculated by dividing the number of instances that are correctly classified as belonging to that class over all the instances that are classified as belonging to that class. For example, the precision of the cluster in the illustrative case is 14/26 = 0.54. Total precision is defined as the average precision of the three classes. Recall is the ratio of correctly classified instances of a class over the total number of instances for this class. If there were to be a total of 38 instances of class label 2 in the experimental surface roughness data, then the recall for class label 2 is 14/38 = 0.39. The total recall is defined as the average for the three classes.

Outlier detection is quantified by comparing the instances that are not part of the 3-cluster partition with actual outliers based on surface roughness labels. The datapoints that are actual outliers and not in the three-cluster partition as classified as true positives (TP) and those that are not actual outliers but are not in the three-cluster partition as false positives (FP). The outlier detection precision is defined as TP/(TP + FP). Clustering results interpreted with these post hoc measures are presented in Table 4, Table 5, Table 6 and Table 7. The results outside the parenthesis is the average of ten independent runs. The standard error over 10 runs is presented in parentheses.

## 6. Discussion

In almost all cases, NC and DBSCAN were the most efficient algorithms as measured by overall accuracy, precision, and recall. The standard error is also smaller in many cases, meaning the results are more stable compared to the other algorithms. Among the feature selection methods, UFSOL was the most efficient method with almost every clustering algorithm. In general, the wrapper models (DGUFS and UFSOL) did better than filter methods (LS and MCFS). Filter methods are less computationally expensive than wrapper methods, and therefore tend to have less predictive power. The spectral clustering algorithm is the only algorithm implemented here that was not resistant to noise (all instances were assigned to one of the three clusters). NC has two parameters that need to be chosen a priori (*λ* assumed be 0.05 and *m* = 2) and DBSCAN also has two parameters (epsilon and minpts), and as such these are easy to tune with little experimentation on a small subset (*n* = 15 in the experiments). NC was marginally better than DBSCAN in identifying the correct outliers.

## 7. Conclusions

A framework to predict the level of surface roughness using data clustering based on features extracted from vibration signals measured during the turning of steel is presented here. The objective was to verify if a certain combination of features cluster into distinct groups using unsupervised clustering and if these clusters relate to surface roughness of the steel samples measured after machining. The study uses four noise-resistant clustering algorithms, including fuzzy clustering, density-based spatial clustering, two versions of kernel clustering, and a generic spectral clustering algorithm. Prior to clustering, the raw feature set was reduced in size using six different feature selection algorithms.

The overarching conclusions are listed below:

1. Among the clustering algorithms used, the noise clustering variant of fuzzy clustering (NC) and density-based spatial clustering with noise (DBSCAN) produced the most accurate partitions that had also high sensitivity and specificity.
2. It was also found that the unsupervised wrapper methods for feature selection when used with unsupervised clustering techniques provided the best subset feature sets.
3. NC was marginally better than DBSCAN in identifying the most probable outliers in measured data. Among the feature selection methods, MCFS, DGUFS, and UFSOL produced the best results.

A comprehensive comparison of various unsupervised clustering methods and feature selection methods for optimal parameter identification has been presented. The absolute values of the identified parameters are immaterial and will change from process to process; however, what is important is the fact that the framework presented here can used in real time to guide the machining process, and if changes need to be made to parameters during processing, parameters can be chosen from the same cluster as the one that corresponds to the “best surface finish”.

## Figures and Tables

**Table 1:** Class labels based on roughness (*R_a_*) values.

Roughness Value (*R_a_*)	Class Attribute	Class Label
*R_a_* ≤ 0.90	Smooth finish	1
0.90 < *R_a_* < 2.50	Medium finish	2
2.50 ≤ *R_a_* ≤ 4.10	Coarse finish	3
*R_a_* > 4.10	Possible outlier	0

**Table 2:** Feature selection methods.

Feature Selection Technique	Method	Class
Relief	Filter	Supervised
Recursive Feature Selection (RFE)	Wrapper	Supervised
Laplacian Score (LS)	Filter	Unsupervised
Multi-Cluster Feature Selection (MCFS)	Filter	Unsupervised
Dependence Guided Unsupervised Feature Selection (DGUFS)	Wrapper	Unsupervised
Unsupervised Feature Selection with Ordinal Locality (UFSOL)	Wrapper	Unsupervised

**Table 3:** List of Features and Comparison of Feature Selection Techniques.

Feature Name	Original Size	ReliefF	RFE	LS	MCFS	DGUFS	UFSOL
Mean	1	1	0	1	0	1	1
Skewness	1	1	1	1	1	0	0
Standard Deviation	1	1	0	1	1	0	0
Kurtosis	1	0	0	0	1	0	0
Variance	1	0	1	0	0	1	1
Crest Factor	1	1	0	1	1	0	0
Peak-to-Peak	1	0	0	1	1	0	1
Root Mean Square (RMS)	1	1	1	1	1	1	0
Root Sum Square (RSSQ)	1	0	0	0	0	0	1
Power Spectral Density	16	8	1	10	12	8	8
Mexican Hat Coefficients	64	12	1	16	16	8	8
Coeflet Wavelet Coefficients	64	12	1	16	16	8	8
Kurtosis of Approximations	10	2	1	1	1	1	0
Skewness of Approximations	10	2	0	0	0	0	1
Kurtosis of Details	10	4	1	4	2	2	2
Skewness of Details	10	2	0	2	2	2	2
RMS of Approximations	10	2	1	1	1	2	2
RMS of Details	10	4	0	4	2	2	2
**Total**	**213**	**53**	**9**	**60**	**58**	**36**	**37**

**Table 4:** Accuracy of clustering algorithms with different feature selection methods.

	All Features	ReliefF	RFE	LS	MCFS	DGUFS	UFSOL
NC	**0.721 (0.029)**	0.687 (0.022)	0.663 (0.019)	0.712 (0.020)	**0.731 (0.017)**	**0.742 (0.018)**	**0.742 (0.018)**
SC	0.609 (0.032)	0.602 (0.025)	0.594 (0.024)	0.654 (0.021)	0.674 (0.019)	0.689 (0.022)	0.691 (0.022)
RBF-KC	0.544 (0.039)	0.546 (0.022)	0.538 (0.022)	0.592 (0.029)	0.612 (0.021)	0.622 (0.023)	0.629 (0.019)
KKM-KC	0.677 (0.024)	0.653 (0.023)	0.677 (0.020)	0.690 (0.022)	0.719 (0.020)	0.722 (0.022)	0.728 (0.0.18)
DBSCAN	0.703 (0.029)	**0.691 (0.021)**	**0.703 (0.019)**	**0.729 (0.018)**	**0.731 (0.017)**	**0.742 (0.018)**	**0.742 (0.018)**

**Table 5:** Precision of clustering algorithms with different feature selection methods.

	All Features	ReliefF	RFE	LS	MCFS	DGUFS	UFSOL
NC	0.619 (0.018)	0.589 (0.009)	**0.573 (0.008)**	0.627 (0.010)	0.633 (0.012)	0.633 (0.009)	0.633 (0.010)
SC	0.554 (0.019)	0.529 (0.011)	0.509 (0.008)	0.563 (0.010)	0.581 (0.013)	0.592 (0.008)	0.600 (0.009)
RBF-KC	0.490 (0.018)	0.483 (0.009)	0.467 (0.002)	0.511 (0.009)	0.520 (0.009)	0.531 (0.012)	0.540 (0.007)
KKM-KC	0.587 (0.019)	0.570 (0.010)	0.537 (0.002)	0.601 (0.008)	0.629 (0.008)	0.658 (0.012)	0.660 (0.008)
DBSCAN	**0.629 (0.019)**	**0.600 (0.010)**	0.564 (0.005)	**0.633 (0.009)**	**0.654 (0.012)**	**0.689 (0.09)**	**0.689 (0.009)**

**Table 6:** Recall of clustering algorithms with different feature selection methods.

	All Features	ReliefF	RFE	LS	MCFS	DGUFS	UFSOL
NC	**0.682 (0.017)**	**0.679 (0.012)**	**0.651 (0.012)**	**0.682 (0.010)**	**0.690 (0.011)**	**0.713 (0.008)**	**0.732 (0.011)**
SC	0.592 (0.018)	0.578 (0.009)	0.552 (0.010)	0.603 (0.009)	0.624 (0.009)	0.638 (0.008)	0.651 (0.012)
RBF-KC	0.527 (0.018)	0.517 (0.010)	0.497 (0.012)	0.534 (0.010)	0.556 (0.008)	0.589 (0.010)	0.610 (0.011)
KKM-KC	0.629 (0.019)	0.592 (0.009)	0.577 (0.007)	0.629 (0.012)	0.629 (0.012)	0.645 (0.010)	0.657 (0.009)
DBSCAN	0.679 (0.020)	0.660 (0.012)	0.629 (0.008)	**0.682 (0.010)**	**0.690 (0.011)**	**0.713 (0.008)**	**0.732 (0.011)**

**Table 7:** Outlier detection precision of clustering algorithms with different feature selection methods.

	All Features	ReliefF	RFE	LS	MCFS	DGUFS	UFSOL
NC	**0.88**	**0.85**	**0.88**	**0.91**	**0.93**	**0.93**	**0.94**
SC	-	-	-	-	-	-	-
RBF-KC	0.85	**0.85**	0.85	0.88	0.88	0.91	0.93
KKM-KC	0.80	0.80	0.80	0.85	0.85	0.91	0.91
DBSCAN	0.85	**0.85**	**0.88**	**0.91**	0.91	**0.93**	0.93

## Data Availability

The data presented in this study are available on request from the corresponding author.

## Abbreviations

NC-FCM	Noise Clustering—Fuzzy c-Means
DBSCAN	Density-Based Spatial Clustering Applications with Noise
RSM	Response Surface Method
NSGA-II	Non-Dominated Sorted Genetic Algorithm II
DF	Desirability Function
FFT	Fast Fourier Transform
DOC	Depth of Cut
RFE	Recursive Feature Selection
LS	Laplacian Score
MCFS	Multi-Cluster Feature Selection
DGUFS	Dependence Guided Unsupervised Feature Selection
UFSOL	Unsupervised Feature Selection with Ordinal Locality
SC	Spectral Clustering
SVC	Support Vector Clustering
KC	Kernel-based Clustering
RBF	Radial Basis Function
KKM	Kernel k-Means
RMS	Root Mean Square
RSSQ	Root Sum Square

## Appendix A

Workpiece Properties: Austenitic Stainless steel (304).

**Table A1:** Composition of 304 Stainless Steel.

Element	Weight %
Carbon (C)	0.07
Chromium (Cr)	18.0
Manganese (Mn)	2.00
Silicon (Si)	1.00
Phosphorous (P)	0.045
Sulphur (S)	0.015
Nickel (Ni)	8.00
Nitrogen (N)	0.10
Iron (Fe)	Balance

**Table A2:** Mechanical Properties of 304 Stainless Steel.

Property	Value
Tensile Strength (annealed)	585 MPa
Ductility	70%
Hardness	70 Rockwell B

**Table A3:** Physical Properties of Stainless Steel.

Property	Value
Density, g/cm^3^	7.93
Melting point	1400–1455 °C
Thermal conductivity (W/m·K)	16.3 (100 °C), 21.5 (500 °C)
Mean coefficient of thermal expansion, (10–6/K)	17.2 (0–100 °C)
